# Paliperidone induced sinus tachycardia in a patient with first episode of psychosis (FEP)

**DOI:** 10.1192/j.eurpsy.2021.2056

**Published:** 2021-08-13

**Authors:** A. Sarafopoulos, D. Antoniadis, V. Karpouza

**Affiliations:** 1 4th Picu, Psychiatric Hospital of Thessaloniki, Thessaloniki, Greece; 2 4th Picu, Psychiatric Hospital of Thessaliniki, Thessaloniki, Greece

**Keywords:** paliperidone, FEP, Tachycardia

## Abstract

**Introduction:**

This is a presentation of the FEP of a 23 y.o. patient. The patient had a Duration of Untreated Psychosis (DUP) of 6 months and Duration of Untreated Illness (DUI) of six years. The therapeutic response and the adverse effects of Paliperidone are being described.

**Objectives:**

To investigate the tolerance of Paliperidone in a patient with FEP.

**Methods:**

The patient was assessed regularly by the psychiatric team consisting of a CT doctor and one General Adult Consultant. Appropriate psychological assessments and investigations took place.

**Results:**

Upon admission the patient appeared guarded. She also presented with weight loss and dehydration. Initial PANSS score was 173, positive subscale 41. The patient was initially treated with monotherapy 6mg of Paliperidone. However, the heart rate was around 100 bpm culminating as high as 156 bpm. The ECG indicated sinus tachycardia. The patient presented with serious EPSs and diarrhea. Simpson-Angus Scale score 10. Metoprolol 25mg was prescribed twice a day. The clinical team proceeded in cross titration replacing Paliperidone with Olanzapine. A brain CT scan was also performed, unremarkable. After 10 days of therapy the PANSS score reduced to 102, positive subscale 21.

**Conclusions:**

Initial sinus tachycardia is a common adverse effect of Paliperidone. However in this case the tachycardia was refractory in time even after the 7th day, making an alternative SGA trial necessary.

**Disclosure:**

No significant relationships.

